# 

**Published:** 1883-12

**Authors:** 


### CONTENTS.

*MISCELLANEOUS.*

PAGE

The I. H. A. and its Work. Editorial, . . . . . . . . 359
Dr. Lippe and the I. H. A. C. Pearson, M. D., . . . . . . 361
The Repetition of the Dose. Ad. Lippe, M. D., . . . . . . 364
Volume Four, . . . . . . . . . . . . . . 367
Potentization. Letter from Dr. Fincke, . . . . . . . 368
Cerebro-Nervous Disorders Peculiar to Women. Thomas Madden Moore, M. D., . . . . . . . . . . . . 372
Note to Subscribers, . . . . . . . . . . . 375
Canned Provisions, . . . . . . . . . . . 376
Hahnemnan the Founder of Isopathy. A Letter from Dr. Swan, . 377
Hahnemann not the Founder of Isopathy. E. J. L., . . . . . 878
The Central New York Homœopathic Society and its Protest against Eclecticism. E. B. Nash, M. D., . . . . . . . . . 378

*CLINICAL BUREAU.*

Dysentery—Lilium Tigrinum. G. M. Pease, M. D., . . . . . 380

*BOOK NOTICES.*

Wesselhœft. The Law of the Similars. Otis Clapp & Son, . . . 381
New Journals: The Medical Era; The Texas Homœopathic Pellet, . 382

*NOTES AND NOTICES.*

Errata—J. Marion Sims—A Second Jenner, . . . . . . . 382

*APPENDIX.*

Cough Repertory, . . . . . . . . . . . . 147–162
Typhoid Fever—Rheumatism, . . . . . . . . . 81–88

